# Involvement of the TNF and FasL Produced by CD11b Kupffer Cells/Macrophages in CCl_4_-Induced Acute Hepatic Injury

**DOI:** 10.1371/journal.pone.0092515

**Published:** 2014-03-25

**Authors:** Atsushi Sato, Hiroyuki Nakashima, Masahiro Nakashima, Masami Ikarashi, Kiyoshi Nishiyama, Manabu Kinoshita, Shuhji Seki

**Affiliations:** 1 Department of Immunology and Microbiology, National Defense Medical College, Namiki, Tokorozawa, Saitama, Japan; 2 Department of Surgery 1, National Defense Medical College, Namiki, Tokorozawa, Saitama, Japan; UAE University, Faculty of Medicine & Health Sciences, United Arab Emirates

## Abstract

We previously reported that F4/80^+^ Kupffer cells are subclassified into CD68^+^ Kupffer cells with phagocytic and ROS producing capacity, and CD11b^+^ Kupffer cells with cytokine-producing capacity. Carbon tetrachloride (CCl_4_)-induced hepatic injury is a well-known chemical-induced hepatocyte injury. In the present study, we investigated the immunological role of Kupffer cells/macrophages in CCl_4_-induced hepatitis in mice. The immunohistochemical analysis of the liver and the flow cytometry of the liver mononuclear cells showed that clodronate liposome (c-lipo) treatment greatly decreased the spindle-shaped F4/80^+^ or CD68^+^ cells, while the oval-shaped F4/80^+^ CD11b^+^ cells increased. Notably, severe hepatic injury induced by CCl_4_ was further aggravated by c-lipo-pretreatment. The population of CD11b^+^ Kupffer cells/macrophages dramatically increased 24 hour (h) after CCl_4_ administration, especially in c-lipo-pretreated mice. The CD11b^+^ Kupffer cells expressed intracellular TNF and surface Fas-ligand (FasL). Furthermore, anti-TNF Ab pretreatment (which decreased the FasL expression of CD11b^+^ Kupffer cells), anti-FasL Ab pretreatment or *gld/gld* mice attenuated the liver injury induced by CCl_4_. CD1d−/− mouse and cell depletion experiments showed that NKT cells and NK cells were not involved in the hepatic injury. The adoptive transfer and cytotoxic assay against primary cultured hepatocytes confirmed the role of CD11b^+^ Kupffer cells in CCl_4_-induced hepatitis. Interestingly, the serum MCP-1 level rapidly increased and peaked at six h after c-lipo pretreatment, suggesting that the MCP-1 produced by c-lipo-phagocytized CD68^+^ Kupffer cells may recruit CD11b^+^ macrophages from the periphery and bone marrow. The CD11b^+^ Kupffer cells producing TNF and FasL thus play a pivotal role in CCl_4_-induced acute hepatic injury.

## Introduction

Carbon tetrachloride (CCl_4_) is a highly toxic chemical agent that induces acute hepatic injury, while chronic administration of CCl_4_ induces fibrosis, cirrhosis and carcinogenesis. Although chronic CCl_4_ injection models have been extensively studied as liver fibrosis and cirrhosis models [1]–[5], the acute phase of this hepatitis has been less characterized. The acute phase of CCl_4_ hepatic injury may be produced by the formation of reactive oxygen species (ROS) in the endoplasmic reticulum of hepatocytes by cytochrome p450 enzymes, which may also induce mitochondrial dysfunction, including changes in calcium homeostasis, energy production and the beta-oxidation of fatty acids, all of which can result in hepatocyte damage [4], [6], [7]. However, although a role for Kupffer cells has been suggested [2], [8]–[10], the immune mechanism involved in the acute phase of CCl_4_-induced hepatic injury has not been extensively examined.

It is now generally accepted that the livers of mice and humans contain various kinds of innate immune cells [11]–[13]. It is well known that liver NK cells and NKT cells potently produce IFN-γ in response to IL-12 and/or LPS [11]–[13]. Interestingly, liver B cells (mostly B-2 cells) produce IL-12 and IFN-γ but not IgM, in response to LPS (vice versa for spleen B cells) [14]. Furthermore, these IL-12-producing liver B cells, in contrast to spleen B cells, phagocytose bacteria and kill them [15], [16]. Therefore, these liver immune cells, including B cells and their cytokines, primarily act as innate immune effectors against infections and tumors by their T helper-1 immune response in the liver. However, they also sometimes induce hepatic injury, septic shock and multi-organ failure [12], [13], [17]. In addition, we have recently reported that liver F4/80^+^ Kupffer cells/macrophages can be subclassified almost exclusively into two different subsets; a CD68^+^ subset with phagocytic, ROS production and bactericidal capacities, and a CD11b^+^ subset with cytokine (TNF and IL-12) production and antitumor capacities [12], [13], [18], [19].

The hepatic injuries induced by α-galactocylceramide (α-GalCer) or bacterial-DNA motifs (CpG-ODN) are TNF/FasL-dependent hepatitis [20]–[23], and concanavalin-A (Con-A)-induced hepatic injury is a TNF/ROS-dependent hepatitis [12], [13], [24]. FasL-expressing NKT cells and ROS-producing CD68^+^ Kupffer cells, both activated by the TNF produced by CD11b^+^ Kupffer cells [17], [20]–[24], are the final effectors in these hepatitis models. CD11b (complement 3b receptor) is present on the surface of monocytes/macrophages, granulocytes and NK cells. CD68 (macrosialin) is also used as a marker of macrophages, including Kupffer cells, and this antigen is also localized in the cytosol of CD11b^+^ macrophages, but it is expressed on the cell surface upon activation [18], [25], [26].

Gadolinium chloride (GdCl_3_) and clodronate liposomes (c-lipo), are both cytotoxic to Kupffer cells, and have been used to deplete Kupffer cells in rodents. Some reports have suggested that GdCl_3_ and c-lipo completely eliminate Kupffer cells based on immunohistochemical examinations. However, we reported and demonstrated herein that these agents deplete only CD68^+^ Kuppfer cells (resident or fixed), but not CD11b^+^ Kupffer cells, based on the flow cytometric analysis of liver mononuclear cells [18], [19]. Consistent with our data, Holt et al. also demonstrated that c-lipo administration did not eliminate the CD11b^high^F4/80^low^ subset, whereas the other CD11b^low^F4/80^high^ subset was almost completely depleted [27]. We consider that the former population corresponds to CD11b^+^ Kupffer cells and the latter population corresponds to CD68^+^ cells in our studies.

In the present study, we demonstrate by immunohistochemistry, as well as flow cytometry, that the large and spindle-shaped CD68^+^ cells were indeed depleted by c-lipo treatment, whereas the small and round-shaped CD11b^+^ population increased. Furthermore, CD11b^+^ Kupffer cells play an important role in the acute phase of CCl_4_-induced hepatitis as a result of their production of TNF and FasL, which occurs in an NKT cell-independent manner. In addition, the c-lipo-phagocytized CD68^+^ Kupffer cells were found to produce monocyte chemoattractant protein (MCP)-1, and lead to the subsequent accumulation of CD11b^+^ Kupffer cells/macrophages into the liver before CCl_4_ injection, which aggravates the hepatic injury induced by CCl_4_ injection.

## Materials and Methods

### Mice and Reagents

The Ethics Committee of Animal Care and Experimentation, National Defense Medical College, Japan, approved all requests for animals and the intended procedures of the present study (Permission number: 12039).

Male C57BL/6 mice (ten weeks of age) and *gld/gld* (*gld*) mice with C57BL/6 background were purchased from Japan SLC (Hamamatsu, Japan). Because B6 CD1d−/− mice were not commercially available, CD1d−/− mice on a BALB/c background were purchased from the Jackson Laboratory. Carbon tetrachloride was purchased from Kanto Chemical Co., Inc. (Tokyo, Japan). C-lipo was purchased from LKT Laboratories, Inc. (St. Paul, MN 55130, USA).

### Induction of Acute Liver Injury

To induce acute liver injury by CCl_4_, mice were injected intraperitoneally with a single dose of CCl_4_ (0.6 mg/kg in oil). Control groups received the same volume of vehicle (oil) intraperitoneally.

### Isolation of MNCs, Including Kupffer Cells

The murine livers were removed under deep anesthesia. The liver MNCs were prepared essentially as described previously [18]. Briefly, the livers were minced and suspended in HBSS containing 0.05% collagenase (Wako, Osaka, Japan), and then were shaken for 20 min in a 37°C water bath. Next, the liver specimens were washed in 1% FBS RPMI 1640 and then filtered through a stainless steel mesh. After mixing in isotonic 33% Percoll solution containing heparin, the samples were centrifuged for 15 min at 500×g at room temperature. After removing the supernatant, the pellets were resuspended in a red blood cell lysis solution and then were washed twice in 10% FBS RPMI 1640.

### Pathological Examination

For pathological examinations, the mice were euthanized prior to removal of their livers. The liver was then immersed in 10% formalin for two days. Slides were prepared from these specimens and stained with hematoxylin and eosin.

### Flow Cytometric Analysis

After incubation with Fc-blocker (2.4 G2; BD PharMingen, San Diego, CA), MNCs were stained with a FITC-conjugated anti-F4/80 Ab (eBioscience, San Diego, CA) [28], Cy5-conjugated anti-CD11b Ab (eBioscience, San Diego, CA) [29] or biotin-conjugated anti-CD68 Ab (FA-11, AbD Serotec, Oxford, UK) [25], [26], [30] with PE-streptavidin. The flow cytometric analysis was performed using an FC500 instrument (Beckman Coulter, Miami, FL).

### Intracellular Staining for TNF

MNCs were incubated with BD GolgiStop (0.7 μg/ml, BD PharMingen) for three h before staining. After incubation with Fc-blocker, the cells were stained with a FITC-conjugated anti-F4/80 Ab and Cy5-conjugated anti-CD11b Ab or a biotin-conjugated anti-CD68 Ab with Cy5-streptavidin. Subsequently, the cells were incubated with BD Cytofix/Cytoperm solution (BD Pharmingen) at 4°C for 20 min and then washed with BD Perm/Wash solution (BD Pharmingen). Thereafter, the cells were stained with a PE-conjugated anti-TNF mAb (eBioscience) or isotype rat IgG1 Ab (eBioscience) at 4°C for 20 min and then analyzed using the FC500 instrument.

### Pretreatment with c-lipo

Clodronate (LKT Laboratories, Inc., St. Paul, MN) was encapsulated into liposomes and 100 μL of a 25 mg/ml suspension was intraperitoneally injected into the mice to deplete the CD68^+^ Kupffer cells 36 h before experiments [31]–[33].

### Neutralization of TNF or FasL, or Depletion of NK or NK/NKT Cells

To neutralize TNF, FasL, anti-TNF Ab (0.5 mg/mouse)(MP6-TX3,BD PharMingen) or anti-FasL Ab (0.5 mg/mouse)(MFL4, BD PharMingen) was injected intravenously one hour before and six h after CCl_4_ administration. To deplete NK cells or both NK and NKT cells, an anti-asialo GM1 (AGM1) Ab (50 μg/mouse) or anti-NK1.1 Ab (PK136; 200 μg/mouse) was injected intravenously into the mice three days before CCl_4_ administration [17], [21], [23]. For the neutralization of FasL in *in vitro* killing assay, 10 μg/ml anti-FasL Ab (MFL4, BD PharMingen) were added to the medium.

### Measurement of the Alanine Amino Transferase, Cytokine and MCP-1 Levels

The serum alanine amino transferase (ALT) level was measured using a DRICHEM 3000V instrument (Fuji Medical Systems, Tokyo). ELISA kits for TNF (BD Biosciences, San Jose, CA, USA) and MCP-1 (R&D system, San Jose, CA, USA) were used to analyze the levels of these cytokines.

### Isolation of F4/80^+^ CD11b^+^ Kupffer Cells from CCl_4_ Treated Mice using MACS Sort System

Livers were obtained from mice 14 h after the injection of CCl_4_, and minced liver specimens without collagenase treatment in 1% FBS RPMI 1640 were filtered through a stainless steel mesh. Thereafter, the liver MNCs were obtained using a 33% Percoll solution. The MNCs were stained with PE-Cy5 labeled anti-F4/80 antibody following conjugation with anti-PE magnetic beads (Miltenyi Biotec GmbH). Beads conjugated F4/80^+^ cells (which were also positive for PE-Cy5) or F4/80^−^ cells were magnetically sorted by Super MACS system (Miltenyi Biotec GmbH).

### Adoptive Transfer Experiments

MACS sorted 5×10^6^ F4/80^+^ cells (PE-Cy5^+^) or 5×10^6^ F4/80^−^ cells were adoptively transferred into normal mice or CD68^+^ Kupffer cell-depleted (by c-lipo) mice. As an experimental control, liver 5×10^6^ F4/80^+^ cells from oil-treated mice were transferred into normal mice. After adoptive transfer, recruitment of transferred F4/80^+^ cells into recipient liver was confirmed by the presence of PE-Cy5 positive cells. The induction of liver injury after transfer was analysed and compared within each group.

### *In vitro* Cytotoxic Assay Against Primary Cultured Hepatocytes

Primary cultured hepatocytes were used as target cells. Hepatocytes were obtained from 8 week of age B6 mice essentially described previously [21]. In brief, liver were perfused with collagenase from portal vein, and dispersed hepatocytes were suspended in hepatocyte growth medium (HCGM) and seeded into collagen type I coated 96 well plate (Iwaki, Funabashi, Japan) with 2.0×10^3^ cells/well concentration. After 12 h of incubation, hepatocytes adhered to the bottom of the plate and medium was changed by HCGM containing 10 μCi of Na_2_^51^Cr0_4_/ml and incubated additional 12 h. The ^51^Cr labeled hepatocytes were washed three times with HCGM, and effector cells were added following 4 h of incubation. F4/80^+^ cells (which were also positive for PE-Cy5) or F4/80^−^ cells were obtained by MACS system as described above. The concentration of effector MNCs were adjusted to 5.0×10^5^ cells/well (250∶1) and 2.5×10^5^ cells/well 125∶1). Culture supernatants were harvested and radio activities were measured by gamma counter.

### Statistical Analysis

The results are expressed as the mean values ± SE. The statistical analyses were performed using a GraphPad Prism 5 software package (GraphPad Software, La Jolla, CA). Statistical evaluations were compared using the standard one-way analysis of variance followed by the Bonferroni post-hoc test. A value of *P*<0.05 was considered to be significant.

## Results

### Depletion of CD68^+^ Kupffer Cells and Aggravation of CCl_4-_ Induced Hepatic Injury by c-lipo Pretreatment

**Figure 1:**
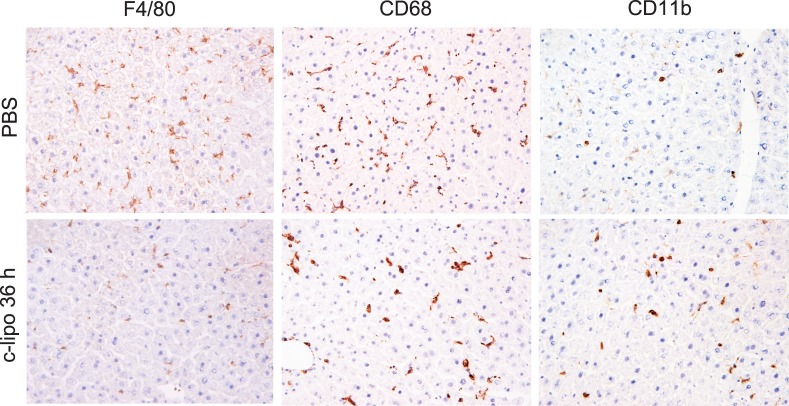
Immunohistochemical staining of liver Kupffer cells/macrophages for F4/80, CD68 and CD11b expression. Livers were harvested from mice 36-lipo or PBS, and liver sections were stained with an anti-F4/80 Ab, anti- CD68 Ab or anti- CD11b Ab. The data are representative of three mice in each group, with similar results obtained for the three animals. ×200 magnification.

**Figure 2:**
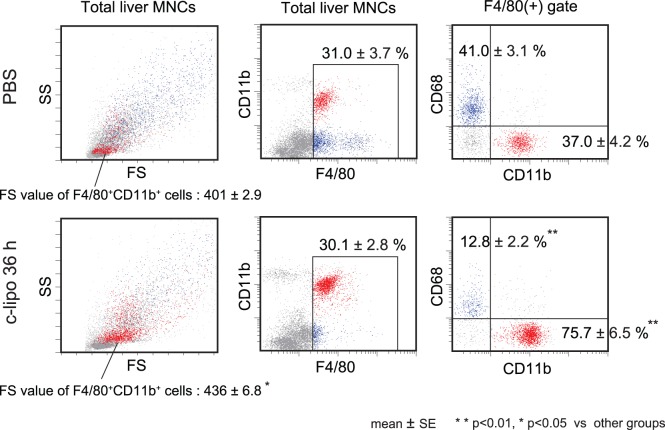
The flow cytometric analysis of Kupffer cells/macrophages. Livers were harvested from mice 36-lipo or PBS, and liver MNCs obtained after collagenase digestion of livers were stained for F4/80, CD11b and CD68. The results of a forward scatter (FS)/side scatter (SS) analysis of the total MNCs are shown (left panels). The mean FS values are also shown (401±2.9 vs. 436±6.8, n = 5, p<005) (left panels). The F4/80/CD11b expression is also presented and the F4/80 positive populations are inside of square gate (middle panels). The CD11b/CD68 expression levels of the gated F4/80-positive cells are demonstrated (right panels). F4/80^+^ CD11b^+^ cells are shown by red dots and the F4/80^+^ CD68^+^ cells are shown by blue dots. **p<0.01, *p<0.05 vs. other groups. The data are representative of five mice in each group, with similar results for the five mice.

We previously reported that c-lipo or GdCl_3_ selectively depleted only CD68^+^ Kupffer cells, but increased the population of CD11b^+^ Kupffer cells/macrophages, as determined by flow cytometry [18]. Indeed, 36 h after c-lipo treatment, an immunohistochemical analysis showed that the spindle-shaped CD68^+^ cells and F4/80^+^ cells were greatly decreased in the liver, while the oval-shaped CD68^+^ or F4/80^+^ cells still remained, and the population of CD11b^+^ cells appeared to increase (Fig. 1). A flow cytometric analysis also confirmed that c-lipo treatment proportionally decreased the liver CD68^+^ Kupffer cells (41% to 12.8%, Fig. 2 right panels) but that the liver CD11b^+^ Kupffer cells/macrophages increased (37% to 75.7%, Fig. 2 right panels). Of note, the number of spleen, bone marrow and peripheral blood CD11b^+^ monocytes/macrophages did not decrease [19]. A forward scatter (FS) and side scatter (SS) analysis revealed that the CD68^+^ Kupffer cells are relatively large and show a complex structure, and most of them disappeared following c-lipo treatment (blue dots, Fig. 2, left panels). In contrast, the CD11b^+^ Kupffer cells/macrophages are small and have a simple structure (red dots, Fig. 2, left panels). The CD11b^+^ Kupffer cells/macrophages became larger after c-lipo treatment, as indicated by the FS analysis (Fig. 2, left panels, FS values; 436±6.8 vs 401±2.9, n = 5, p<0.05), suggesting that they were activated after c-lipo treatment. However, the total number of liver MNCs yielded from the liver did not significantly change following the c-lipo treatment (approximately 7×10^6^/liver). Thus, the number of CD68^+^ Kupffer cells decreased, while the number of CD11b^+^ Kupffer cells/macrophages increased, in the liver.

**Figure 3:**
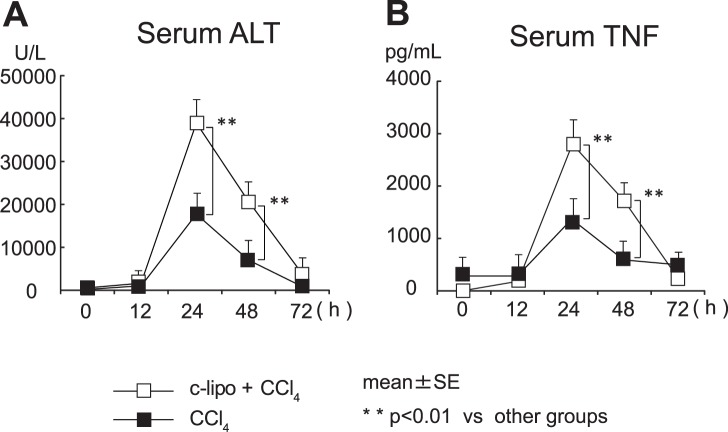
C-lipo pretreatment aggravates CCl_4-_induced hepatic injury. Mice pretreated with c-lipo or PBS were injected intraperitoneally with CCl_4_ or oil. (A) The serum ALT levels were evaluated at the indicated times after CCl_4_ stimulation. (B) The influence of CCl_4_ challenge on the serum TNF levels. The data are the means ± SE from 10 mice in each group. **p<0.01 vs. other groups.

CCl_4_ injection induced severe hepatic injury, as indicated by the ALT levels, and the hepatic injury was aggravated in c-lipo pretreated mice (Fig. 3A). Consistently, the TNF levels in c-lipo pretreated mice after CCl_4_ injection were higher than those of control mice (Fig. 3B).

### The Increase in the Number of F4/80^+^ CD11b^+^ Kupffer Cells After CCl_4_ Injection in Mice with or without c-lipo Pretreatment, and the Liver Histopathology

**Figure 4:**
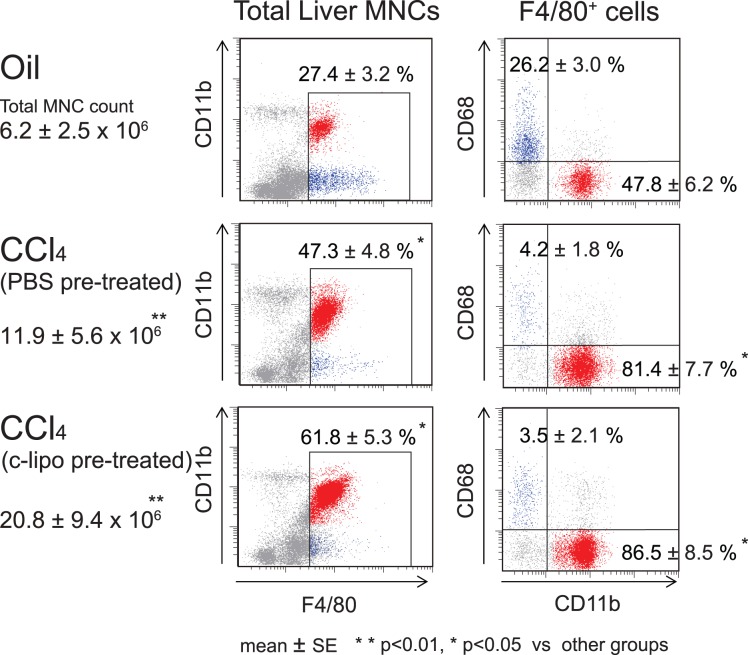
The effect of c-lipo pretreatment on the population of F4/80^+^ CD11b^+^ Kupffer cells after CCl_4_ injection. The changes in the population of F4/80-positive Kupffer cells after CCl_4_ challenge with c-lipo or PBS pretreatment were examined. Mice were intraperitoneally injected with CCl_4_ 36 h after c-lipo or PBS treatment. As an experimental control, the vehicle oil was intraperitoneally injected. The changes in the total amount of liver MNCs, the population of F4/80^+^ cells, and the proportion of each Kupffer cell subset following CCl_4_ challenge after c-lipo or PBS pretreatment are shown. The F4/80^+^ CD11b^+^ cells are shown by red dots and the F4/80^+^ CD68^+^ cells are shown by blue dots. The data are representative of five mice in each group, with similar results. **p<0.01, *p<0.05 vs. other groups.

**Figure 5:**
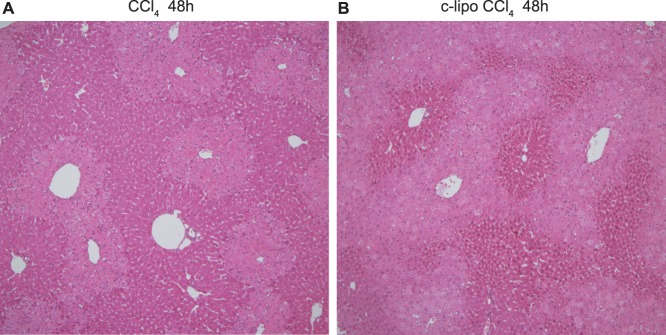
The effect of c-lipo pretreatment on the liver histopathology after CCl_4_ injection. The pathological findings of c-lipo- or PBS-pretreated mice 48 h after CCl_4_ challenge. The liver is shown (×100 magnification; H&E staining). (A) The PBS pretreated mice and (B) c-lipo pretreated mice. The data are representative of three mice in each group, with similar results observed for all animals.

Twenty-four h after CCl_4_ injection, the population of F4/80^+^ CD11b^+^ Kupffer cells was greatly increased compared to that in control oil-injected mice, and c-lipo-pretreatment further increased the number of F4/80^+^ Kupffer cells after CCl_4_ injection (Fig. 4). However, the number of CD68^+^ Kupffer cells was reduced, especially in c-lipo-pretreated mice, after CCl_4_ injection. In addition, the livers of c-lipo-pretreated mice showed more and larger necrotic areas after CCl_4_-injection than did the PBS-pretreated control mice (Fig. 5).

### The Role of TNF, FasL, NKT Cells and NK Cells in CCl_4_-induced Hepatic Injury

**Figure 6:**
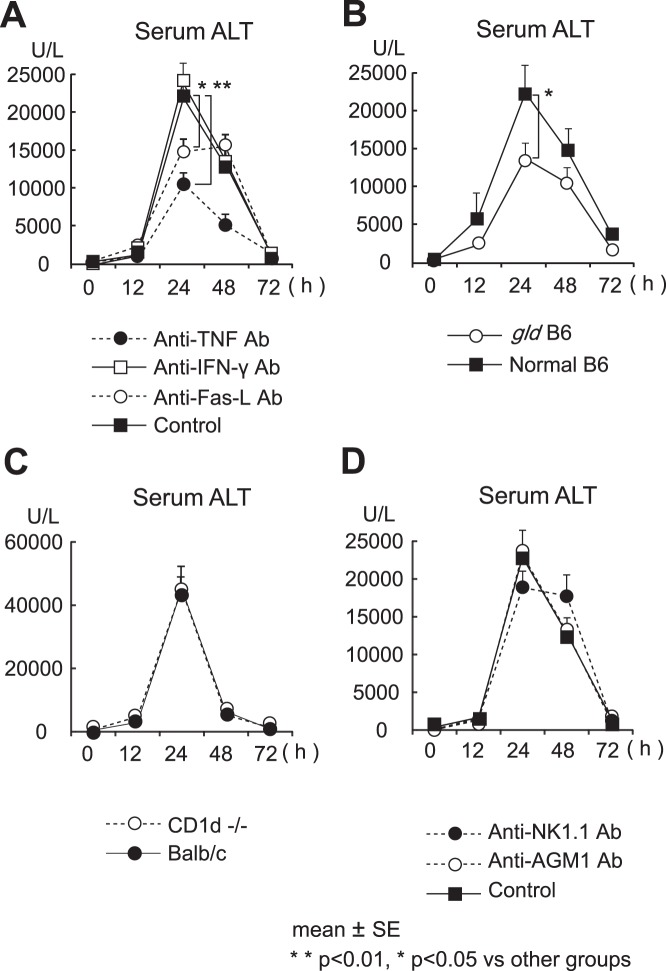
The role of TNF, FasL, NKT cells and NK cells in CCl_4_-induced hepatic injury. (A) The serum ALT levels after CCl_4_ administration in mice pretreated with an anti-TNF Ab, IFN-γ or anti-FasL Ab, (B) in FasL deficient *gld* mice, (C) in NKT cell-deficient CD1d−/− mice, and (D) in mice pretreated with an anti-AGM1 Ab or anti-NK1.1 Ab at the indicated time points. The data are the means ± SE from 10 mice in each group. **p<0.01, *p<0.05 vs. other groups.

Since we previously reported that TNF and/or FasL are involved in the pathogenesis of some experimental models of hepatitis [17], [21], [23], we examined the functions of TNF and FasL in CCl_4_-induced hepatitis. Pretreatment of mice with a neutralizing anti-TNF Ab or anti-FasL Ab significantly decreased the serum ALT levels after CCl_4_ administration (Fig. 6A). However, pretreatment with a neutralizing anti-IFN-γ Ab did not affect the serum ALT levels after CCl_4_ administration (Fig. 6A). Additionally, liver injury was significantly ameliorated in FasL deficient *gld* mice (Fig. 6B). These results suggest that TNF and FasL, but not IFN-γ, are profoundly involved in the CCl_4-_induced acute hepatic injury.

Since we previously reported that NKT cells are responsible for the hepatic injury induced by α-GalCer or bacterial DNA motifs [17], [21], [23], we next examined the effects of CCl_4_ on the CD1d−/− mice. Since CD1d−/− mice on a B6 background were not commercially available, we used CD1d−/− mice on a BALB/c background. The results showed that the serum ALT levels were not significantly different between CD1d−/− mice and control mice (Fig. 6C). Next, we examined the effect of CCl_4_ on mice depleted of NK cells by an anti-AGM1 Ab, or depleted of NKT cells as well as NK cells (by an anti-NK1.1 Ab), and found that the serum ALT levels were similar to those in control mice (Fig. 6D). These results suggest that NK/NKT cells are not involved in the CCl_4_-induced acute hepatic injury.

### Intracellular TNF Production and FasL Expression of Kupffer Cells, and the Effects of an anti-TNF Ab

**Figure 7:**
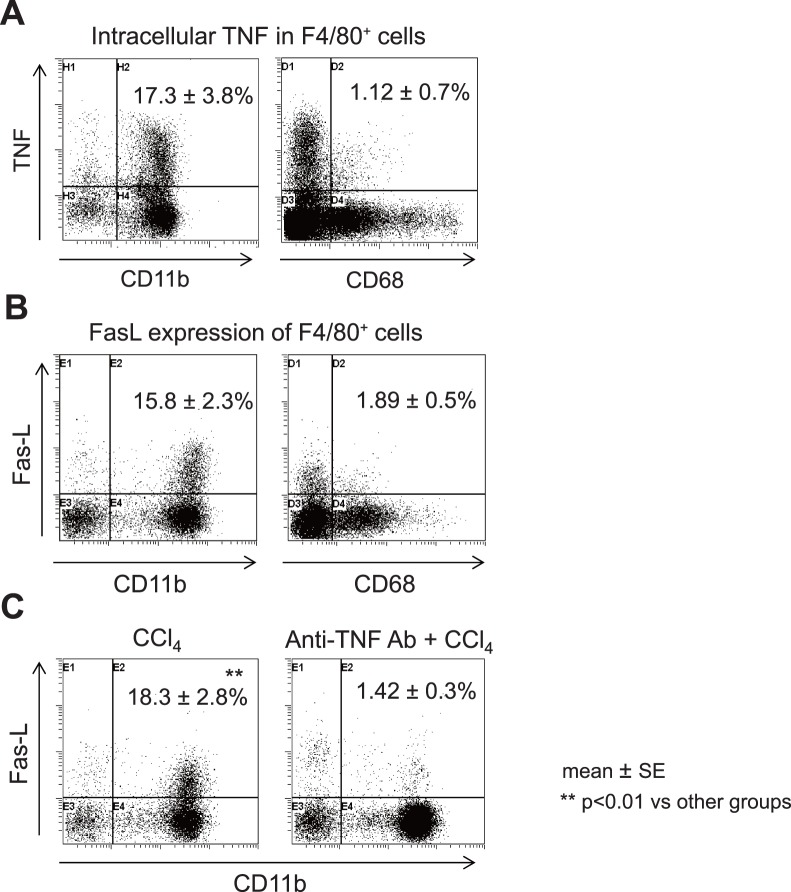
The intracellular TNF production and FasL expression of Kupffer cells. (A) The expression of intra-cellular TNF and (B) FasL expression in the F4/80^+^ CD11b^+^ cells or F4/80^+^ CD68^+^ cells was examined 12 h after the CCl_4_ injection. (C) The effect of pretreatment with a neutralizing TNF Ab on the FasL expression of F4/80^+^ CD11b^+^ cells. The data are representative of five mice in each experiment, with similar results obtained for each mouse. **p<0.01 vs. other groups.

We next examined the intracellular TNF production and surface FasL expression of liver MNCs 12 h after CCl_4_ injection. The F4/80^+^ CD11b^+^ Kupffer cells, but not other cells, including F4/80^+^ CD68^+^ Kupffer cells, produced TNF and expressed FasL (Figs. 7A, B). The staining of F4/80^+^ CD11b^+^ Kupffer cells for TNF was lower at six and 24 h after CCl_4_ injection compared to that at 12 h after CCl_4_ injection (not shown). We further examined the relationship between the TNF and FasL expression of F4/80^+^ CD11b^+^ Kupffer cells. Pretreatment with a neutralizing TNF Ab dramatically decreased the FasL expression of the F4/80^+^ CD11b^+^ Kupffer cells (Fig. 7C). These results suggest that the TNF produced by F4/80^+^ CD11b^+^ Kupffer cells induces their FasL expression, and that F4/80^+^ CD11b^+^ Kupffer cells play a crucial role in CCl_4_-induced acute hepatic injury via TNF/FasL.

### Induction of Hepatic Injury in Mice by the Adoptive Transfer of CD11b^+^ Kupffer Cells from mice Injected with CCl_4_

**Figure 8:**
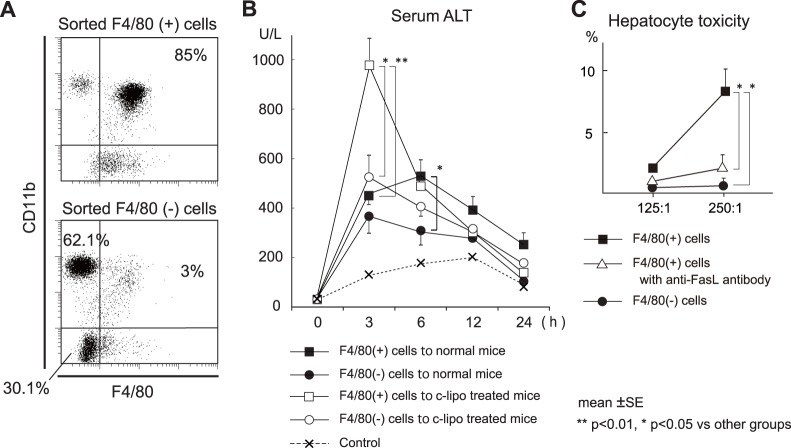
The induction of hepatic injury in mice adoptively transferred mice with CD11b^+^ Kupffer cells from mice injected with CCl_4_, and in vitro cytotoxic assay against primary cultured hepatocytes. Livers were obtained from mice 14_4_. Minced liver specimens without collagenase treatment in 1% FBS RPMI 1640 were filtered through a stainless steel mesh. Thereafter, the liver MNCs were obtained using a 33% Percoll solution. The liver MNCs were subjected to MACS sorting to separate the F4/80^+^ cells and F4/80^−^ cells. (A) The purity of the MACS-sorted F4/80^+^ cells and F4/80^−^ cells was confirmed by flow cytometry. The harvested F4/80^+^ cells were mostly CD11b^+^ Kupffer cells. (B) 5×10^6^ F4/80^+^ cells or 5×10^6^ F4/80^−^ cells were adoptively transferred into normal mice or CD68^+^ Kupffer cell-depleted mice (by c-lipo), and the serum ALT levels were evaluated at the indicated time points. As an experimental control, 5×10^6^ liver F4/80^+^ cells from oil-treated mice were transferred into normal mice. (C) Cytotoxic activity of sorted F4/80^+^ cells and F4/80^−^ cells against primary cultured hepatocytes. The data are the means ± SE from six mice in each group. **p<0.01, *p<0.05 vs. other groups. Cytotoxic activity of F4/80^+^ cells with anti-FasL antibody was also measured. The data are the means ± SE from three independent experiments. *p<0.05 vs. other groups.

We previously reported that the hepatic MNCs obtained without collagenase digestion contain many CD11b^+^ Kupffer cells but few CD68^+^ Kupffer cells [18]. Consistently, the MACS-sorted liver F4/80^+^ cells without collagenase digestion from CCl_4_-injected mice were primarily F4/80^+^ CD11b^+^ Kupffer cells (85%, Fig. 8A). A major proportion of the F4/80^-^ CD11b high cells were Gr1-positive neutrophils (62%, Fig. 8A and not shown) and the CD11b^-^ F4/80^−^ cells were lymphocytes (30%, Fig. 8A and not shown). Moreover, MACS sorted F4/80^+^ CD11b^+^ Kupffer cells were adoptively transferred into normal mice or CD68^+^ cell depleted mice, and serum ALTs were examined. The hepatic injury induced by transferred F4/80^+^ CD11b^+^ Kupffer cells was stronger than that induced by transferred F4/80^−^ cells (Fig. 8B). Moreover, when F4/80^+^ CD11b^+^ Kupffer cells from CCl_4_-injected mice were transferred into mice depleted of CD68^+^ Kupffer cells (36 h after c-lipo injection), a more severe hepatic injury was evoked than that in mice without c-lipo pretreatment (Fig. 8B). These results raise the possibility that resident CD68^+^ Kupffer cells may normally inhibit the function of CD11b^+^ Kupffer cells/macrophages. However, it should be noted that the transfer of liver F4/80^−^ cells also induced a substantial hepatic injury in clodronate-pretreated mice (Fig. 8B), suggesting that the activated neutrophils contained in the F4/80^−^ cells may also have hepatotoxicity under *in vivo* condition. Flow cytometric analysis of recipient liver MNCs at 1.5 h after the adoptive transfer without additional staining showed that 4.5% were positive for PE-Cy5 in mice transferred with F4/80^+^ cells (Fig. S1, left panel) and few (0.6%) were postive in mice transferred with F4/80^−^ cells (non-specific staining) (Fig. S1, middle panel). FS/SS analysis verified that such positive cells (in left panel) were confirmed to be macrophages (Fig. S1, right panel). Furthermore, MACS sorted F4/80^+^ CD11b^+^ Kupffer cells from CCl_4_-injected mice showed cytotoxicity against primary cultured hepatocytes *in vitro*, and this cytotoxicity was effectively blocked by neutralization of FasL, whereas F4/80^−^ cells did not showed the killing activity *in vitro* (Fig. 8C).

### C-lipo Treatment of Mice Before CCl_4_ Administration Increases the Serum Level of MCP-1

**Figure 9:**
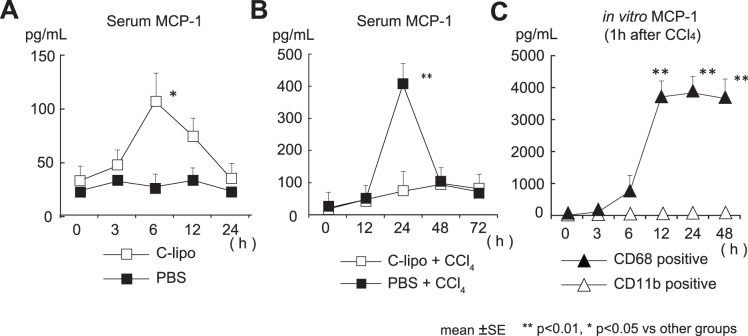
The serum MCP-1 levels after c-lipo treatment and the MCP-1 production from CD68^+^ Kupffer cells after CCl_4_ injection with/without c-lipo pretreatment. (A) There was an increase in the serum MCP-1 levels in mice early after c-lipo injection. The mice were i.p. injected with c-lipo or PBS, the sera were obtained at the indicated time points, and the MCP-1 levels were measured (n = 3 in each group). (B) The serum MCP-1 levels did not increase after CCl_4_ injection in mice pretreated with c-lipo. The mice pretreated with c-lipo or PBS were injected intraperitoneally with CCl_4_ or oil, and sera were obtained at the indicated time points to measure the MCP-1 levels (n = 3 in each group).(C) The *ex vivo* production of MCP-1 from the liver CD68^+^ Kupffer cells from CCl_4_-injected mice. One hour after the injection of CCl_4_, the liver MNCs were harvested from the liver by collagenase treatment, and CD68^+^ cells were obtained by magnetic beads (MACS system). F4/80^+^ CD11b^+^ cells obtained by F4/80 magnetic beads from the liver MNCs from c-lipo treated mice. Both purified subsets were cultured *in vitro* for the indicated amounts of time. The data are the means ± SE from three independent experiments. **p<0.01, *p<0.05 vs. other groups.

To elucidate the mechanism by which CD11b Kupffer cells are increased by c-lipo pretreatment, the serum MCP-1 levels were monitored after c-lipo injection. MCP-1 is a major chemokine, and is a ligand for CC-chemokine receptor 2 (CCR2). Intriguingly, the serum MCP-1 levels rapidly increased and peaked at six h after c-lipo injection after c-lipo treatment (Fig. 9A). Furthermore, the MCP-1 levels did not increase any more in the c-lipo-pretreated mice after CCl_4_ injection (Fig. 9B). These results suggest that the CD68 Kupffer cells are activated after phagocytosing c-lipo, and that they produced MCP-1 and thereafter underwent apoptosis due to the cytotoxicity of clodronate. Our results also suggest that MCP-1 plays a critical role in the recruitment and activation of CD11b Kupffer cells from the periphery or bone marrow.

To confirm the presence of MCP-1-producing CD68 cells, liver MNCs were harvested from mice one hour after CCl_4_ administration, and CD68^+^ cells were purified by the MACS system from liver MNCs obtained from liver specimens with collagenase treatment, and F4/80^+^ CD11b^+^ cells (F4/80^+^ cells from mice depleted of CD68 cells by c-lipo) were also obtained by the MACS system. Both subsets were cultured *in vitro* for the indicated amount of time. The results showed that CD68 cells produced a substantial amount of MCP-1 beyond 12 h after culture, but CD11b cells did not produce any MCP-1 (Fig. 9C). We also found that, in mice depleted of CD68 Kupffer cells injected with viable *Staphylococcus aureus,* the serum MCP-1 levels (peak at 3 h) were greatly reduced compared to those in control mice, and that CD68 Kupffer cell-depleted liver MNCs cultured with bacteria *in vitro* did not produce any MCP-1 [19]. However, it was considered possible that the MCP-1 produced by CD68 Kupffer cells at 24 h after CCl_4_ administration may not be involved in the recruitment of CD11b macrophages/Kupffer cells or hepatic injury.

## Discussion

In the current study, we explored a unique immunological mechanism of CCl_4_-induced acute hepatic injury in mice. Namely, CD11b^+^ Kupffer cells produce TNF, as well as FasL, and induce hepatic injury, in which IFN-γ, NK cells and NKT cells are not involved. Furthermore, c-lipo pretreatment to deplete CD68^+^ Kupffer cells promoted MCP-1 production from the CD68^+^ Kupffer cells, presumably before they underwent apoptosis, which led to the accumulation and activation of CD11b^+^ Kupffer cells, and markedly aggravated the hepatic injury following CCl_4_ injection. Both CpG-ODN and α-GalCer-mediated hepatitis are TNF/FasL/Fas pathway-dependent, and the final effectors in these types of hepatitis are FasL-expressing NKT cells activated by the TNF produced by CD11b^+^ Kupffer cells/macrophages [17], [21], [23], [34]. However, the final immune effectors in CCl_4_-induced hepatitis are CD11b^+^ Kupffer cells/macrophages, which themselves have FasL expression.

We recently demonstrated that CD68^+^ Kupffer cells are fixed Kupffer cells and cannot be harvested unless collagenase treatment of liver tissues is carried out, whereas CD11b^+^ Kupffer cells are easily obtained without collagenase treatment from liver specimens [18]. In addition, although CD68^+^ Kupffer cells are mainly located in the mid-zonal region between the portal vein and the central vein, CD11b^+^ Kupffer cells are equally distributed in the liver tissues [18]. Therefore, it was suggested that CD68^+^ cells are resident Kupffer cells, and that CD11b^+^ cells may be recruited from the periphery or bone marrow to the inflamed liver [18], [19]. Furthermore, the functions of these cell subsets are quite different: CD68^+^ Kupffer cells have phagocytic, ROS-producing and bactericidal activities, while CD11b^+^ Kupffer cells have cytokine (IL-12 and TNF)-producing capacity and are involved in antitumor immunity [18], [19]. Our present results also confirmed by immunohistochemistry, as well as flow cytometry, that c-lipo depletes CD68^+^ Kupffer cells, but increases the number of CD11b^+^ Kupffer cells. Furthermore, resident CD68^+^ Kupffer cells are radio-resistant, whereas CD11b^+^ Kupffer cells/macrophages are radio-sensitive [19], [27] which were reconstituted by the transfer of bone marrow cells [19]. Therefore, they are distinct types of macrophages, and most of the monocytes/macrophages in the spleen and peripheral blood are CD11b^+^ cells, while CD68^+^ Kupffer cells predominate in the liver. Although intracellular CD68 was present in the cytosol of CD11b^+^ Kupffer cells and may be expressed on the cell surface of the cells upon activation [26], the intra-cellular CD68 expression was still much lower than that of CD68^+^ Kupffer cells, as revealed by flow cytometry (our unpublished observation).

In contrast to the hepatic injury induced by either α-GalCer or CpG-ODN, CD68^+^ Kupffer cells and their ROS production induced by the TNF produced by CD11b Kupffer cells/macrophages are the final effectors in Con-A-induced hepatitis [12], [13], [24]. In all of these types of hepatitis, the TNF was produced by CD11b^+^ Kupffer cells in the early period (at 1 h) after the injection of reagents [17], [20]–[23]. However, the serum TNF levels did not start to increase until 12 h after CCl_4_ injection, and the intracellular production of TNF in CD11b^+^ Kupffer cells reached a maximum at 12 h after CCl_4_ injection, suggesting that TNF may be released from CD11b^+^ Kupffer cells into the systemic circulation beyond 12 h after CCl_4_ injection. These results imply that, although CD11b^+^ Kupffer cells are effectors involved in the hepatic injury induced by CCl_4_, CD11b^+^ Kupffer cells may produce TNF and FasL in order to reject the hepatocytes chemically-damaged by CCl_4_ to ensure the early termination of hepatic injury. Karlmark et al. also pointed out the marked infiltration of F4/80^+^ CD11b^+^ Gr1^+^ macrophages in the acute phase of CCl_4_ hepatic injury, and demonstrated the indispensable role of CCR2 (MCP-1 ligand) for their recruitment to the liver [2]. It is plausible that the CD11b^+^ Kupffer cells in our study may be identical to the population reported in their study.

It was previously reported that CCR2-deficient mice showed a dramatic reduction in macrophage accumulation in both the peritoneal cavity and liver upon exposure to inflammatory stimuli [2], [35], [36]. MCP-1 is a major ligand of CCR2 [36], and is thus an important chemokine that recruits monocytes/macrophages to inflamed organs and tissues. CD68 Kupffer cells may be activated by phagocytosing c-lipo, and may produce MCP-1 before undergoing apoptosis. Although CD68 Kupffer cell-depleted mice did not show an increase in MCP-1 after CCl_4_ injection, the hepatic injury, as well as the CD11b^+^ cell recruitment, was enhanced 24 h after CCl_4_ injection. These results suggest that, although increased MCP-1 in the early phase (6 h after c-lipo-pretreatment) may be required to increase and prime CD11b Kupffer cells/macrophages in the liver before CCl_4_ administration, other cytokines, including TNF, may be critical for further recruitment of CD11b^+^ cells into the liver and for the resultant hepatic injury after CCl_4_ injection.

The time course of the serum MCP-1 levels after the injection of c-lipo or bacteria [19] suggests an important point. When CD11b Kupffer cells are activated and produce TNF *in vivo* due to exposure to LPS, CpG-ODN and α-GalCer or whole bacteria, the serum TNF levels usually peak at 1 h after administration [17], [22], [23], [37], which is much earlier than the peaks of serum MCP-1 after bacteria or c-lipo injection (3 h or 6 h, respectively). This may be because the production of MCP-1 from CD68 Kupffer cells requires the digestion of c-lipo or bacteria (phagolysosomal formation) [19], whereas CD11b Kupffer cells may rapidly respond to these ligands via TLR-4, 9, or CD1d.

However, further investigations of both CD68^+^ Kupffer cells and CD11b^+^ Kupffer cells/macrophages and their mutual interactions, as well as their interactions with other liver leukocytes, are required for understanding the chronic inflammation and fibrosis induced by CCl_4_.

## Supporting Information

Figure S1 **The recruitment of adoptive transferred F4/80^+^ cells into liver.** Liver MNCs were isolated from CCl_4_ treated mice and stained with PE-Cy5 labeled anti-F4/80 antibody. F4/80^+^ and F4/80^−^ cells were separated using anti-PE magnetic beads and MACS sorting device. Sorted F4/80^+^ cells labeled with PE-Cy5, and F4/80^−^ cells without labelling, were adoptively transferred into normal mice. Then, liver MNCs obtained from each recipient mouse at 1.5 h after adoptive transfer were analyzed by flow cytometry without additional staining. PE-Cy5 (F4/80) positive cells were counted and depicted in red area and dots. Data are representative of three mice in each group, with similar results.
(EPS)
