# Correction: Involvement of the TNF and FasL Produced by CD11b Kupffer Cells/Macrophages in CCl_4_-Induced Acute Hepatic Injury

**DOI:** 10.1371/journal.pone.0114065

**Published:** 2014-11-13

**Authors:** The PLOS ONE Staff

There is an error in the first sentence of the “Induction of Acute Liver Injury” subsection of the Materials and Methods. The correct sentence is: To induce acute liver injury by CCl_4_, mice were injected intraperitoneally with a single dose of CCl_4_ (0.6 ml/kg in oil).
